# Skeletal Muscle Regeneration and Oxidative Stress Are Altered in Chronic Kidney Disease

**DOI:** 10.1371/journal.pone.0159411

**Published:** 2016-08-03

**Authors:** Keith G. Avin, Neal X. Chen, Jason M. Organ, Chad Zarse, Kalisha O’Neill, Richard G. Conway, Robert J. Konrad, Robert L. Bacallao, Matthew R. Allen, Sharon M. Moe

**Affiliations:** 1 Department of Physical Therapy, Indiana University School of Health and Rehabilitation Sciences, Indianapolis, IN, United States of America; 2 Division of Nephrology, Indiana University School of Medicine, Indianapolis, IN, United States of America; 3 Department of Anatomy & Cell Biology, Indiana University School of Medicine, Indianapolis, IN, United States of America; 4 Lilly Research laboratories, Eli Lilly and Company, Indianapolis, IN, United States of America; Universidade de Sao Paulo, BRAZIL

## Abstract

Skeletal muscle atrophy and impaired muscle function are associated with lower health-related quality of life, and greater disability and mortality risk in those with chronic kidney disease (CKD). However, the pathogenesis of skeletal dysfunction in CKD is unknown. We used a slow progressing, naturally occurring, CKD rat model (Cy/+ rat) with hormonal abnormalities consistent with clinical presentations of CKD to study skeletal muscle signaling. The CKD rats demonstrated augmented skeletal muscle regeneration with higher activation and differentiation signals in muscle cells (i.e. lower Pax-7; higher MyoD and myogenin RNA expression). However, there was also higher expression of proteolytic markers (Atrogin-1 and MuRF-1) in CKD muscle relative to normal. CKD animals had higher indices of oxidative stress compared to normal, evident by elevated plasma levels of an oxidative stress marker, 8-hydroxy-2' -deoxyguanosine (8-OHdG), increased muscle expression of succinate dehydrogenase (SDH) and Nox4 and altered mitochondria morphology. Furthermore, we show significantly higher serum levels of myostatin and expression of myostatin in skeletal muscle of CKD animals compared to normal. Taken together, these data show aberrant regeneration and proteolytic signaling that is associated with oxidative stress and high levels of myostatin in the setting of CKD. These changes likely play a role in the compromised skeletal muscle function that exists in CKD.

## Introduction

Chronic kidney disease (CKD) is a progressive disease that leads to increased inflammation, increased concentrations of detrimental uremic toxins, augmented hormonal status and an impaired musculoskeletal system [1]. These musculoskeletal deficits contribute to a lower health-related quality of life, greater disability, and reduced physical activity associated with increased risk of mortality [2–4]. Physical deficits associated with CKD are due in part to both muscle loss (atrophy) and muscle weakness [5, 6]. Unfortunately, little is known about the cellular mechanisms underlying skeletal muscle changes in CKD.

Muscle dysfunction in CKD may be accelerated by either increased catabolism, decreased protein synthesis or impaired regeneration. However, it is not clear which are the overriding factors that sway the balance between muscle production and loss in CKD. A number of studies and reviews postulate oxidative stress as a major contributor of muscle atrophy [7, 8]. Oxidative stress is the result of accumulated endogenous reactive oxygen species (ROS); ROS can amass from dysfunctional mitochondria and increased NADPH oxidases (NOX). Specifically, oxidative stress can lead to atrophy by activating autophagy pathways through forkhead transcription factor (FoxO) 3-mediated transcription factors, Atrogin-1 and muscle ring finger protein 1 (MuRF-1) [9, 10]. Oxidative stress may also affect skeletal muscle through the myostatin pathway [11]. Myostatin, (growth differentiation factor 8) regulates muscle atrophy via activation of proteolytic pathways and impaired muscle regeneration [12]. Muscle regeneration is an organized process that, in response to a harmful stimulus, activates quiescent muscle stem (satellite) cells to differentiate and form myotubes and subsequent myofibers [13]. Impaired regenerative processes and increased catabolism have been studied in mouse models of kidney injury [14]. However, it is not clear how these processes may be altered in a slow progressing, naturally occurring CKD model, which may better capture the progressive nature of human CKD.

We have previously published data demonstrating that by 35 weeks of age, the Cy/+ (CKD) rat has developed progressive, significant azotemia, hyperphosphatemia, secondary hyperparathyroidism, and markedly elevated FGF23, which result in kidney function equal to approximately 15% of kidney function in the normal littermates (NLs) [15]. We recently published that CKD rats demonstrate significantly reduced muscle fiber cross sectional area indicative of atrophy and peak isometric torque during ankle dorsiflexion [16]. In the current study, we tested the hypothesis that in CKD there is increased oxidative stress and myostatin levels that together could explain altered skeletal muscle regeneration and catabolic signaling.

## Methods

### Animal model and tissue harvest

We used a naturally occurring rat model of Chronic Kidney Disease-Mineral Bone Disorder (CKD-MBD); the Cy/+ rat model (CKD rat) transmits cystic kidney disease as an autosomal dominant trait with slow progressing CKD due to a missense mutation in the gene Anks6 (samcystin) [17]. The CKD rat with slowly progressive azotemia results in terminal uremia by 40 weeks and development of all three manifestations of CKD-MBD (i.e. biochemical abnormalities, extraskeletal calcification, and abnormal bone) [15]. Weaned rats were housed in open top, shoebox cages, and had free access to tap water and standard chow until they were 24 weeks old when they were switched from a standard pellet rat chow to a diet of 18% casein-based protein, 0.7% phosphorus, 0.7% calcium, 5% fat (Harlan Teklan TD.04539), until sacrifice, which leads to more reproducible phenotype [18]. Male Cy/+ rats (hereafter called CKD rat), and normal littermates (NL) rat; (n = 6–8 each group) were sacrificed at 35 weeks with pentobarbital (50 mg/kg intraperitoneal) and blood was collected for oxidative stress markers and myostatin assays. The extensor digitorum longus (EDL) was collected and stored at –80°C for RNA and protein isolation. To preserve the middle third of the left EDL for histology, the muscle was placed on a piece of corkboard, in optimal cutting temperature compound (OCT) and frozen in liquid nitrogen chilled 2-methylybutane for 45 seconds; then stored at -80°C until analysis. All procedures were reviewed and approved by the Indiana University School of Medicine Institutional Animal Care and Use Committee, which adheres to the Guide for the Ethical Treatment of Animals to minimize pain and suffering. (http://grants.nih.gov/grants/olaw/Guide-for-the-Care-and-Use-of-Laboratory-Animals.pdf)

### RNA isolation and real time PCR

Total RNA from normal and CKD EDL was isolated using miRNeasy Mini Kit (Qiagen) according to the manufacturer’s instruction. Total RNA was eluted from the column in RNase-free water and stored at –80°C. The gene and miRNA expression was determined by real time PCR using TaqMan miRNA assays (Applied Biosystems, Foster City, CA). Target-specific PCR primers (Pax-7, MyoD, Myostatin, Myogenin, Atrogin 1, MuRF-1, miR-29b, Activin 2b, SOD-1, and SOD-2) were obtained from Applied Biosystems. Real-time PCR amplifications were performed using TaqMan miRNA Assays (TaqMan MGP probes, FAM dye-labeled) using Applied Biosystems ViiA 7 Real-Time PCR systems (Applied Biosystems). The cycle number at which the amplification plot crosses the threshold was calculated (CT), and the ΔΔCT method was used to analyze the relative changes in gene expression and normalized by β-actin or U6 (RNA and miRNA, respectively).

### Western blot

Western blotting was performed as previously described [19]. In brief, the EDL from NL and CKD were homogenized and the total tissue protein lysates were stored at -20C. The expression of Nox4 was measured using antibody against Nox4 (1:300 dilution; Novus Biologicals, Littleton, CO). Nuclear and cytosolic protein was isolated using Cayman’s Nuclear Extraction kit (Cayman Chemical Company, Ann Arbor, MI) according to the manufacturer’s instructions. Nrf2 was measured in the nuclear fraction, and the major regulator of Nrf2, Keap1 (Kelch-like ECH-associated protein I) was measured in the cytosolic fraction with antibody against Nrf2 or Keap 1(1:1000, Santa Cruz Biotechnology, Santa Cruz, CA) overnight at 4°C followed by incubating with peroxidase conjugated secondary antibody (1:5000 dilution), and immunodetection with the Enhanced Chemiluminescence Prime Western Blot Detection Reagent (Amersham, Piscataway, NJ). The band intensity was analyzed by ChemiDoc MP Imaging System (Imaging Lab 4.0, Bio-Rad, Richmond, CA) and normalized to total protein expression using Ponceau S (Santa Cruz Biotechnology, Santa Cruz, CA).

### Plasma oxidative stress assay and myostatin assay

Plasma levels of an oxidative stress marker, 8-hydroxy-2' -deoxyguanosine (8-OHdG), were measured using a DNA damage ELISA kit (Enzo Life Sciences, Farmingdale, NY). Plasma myostatin levels were measured using a dual-monoclonal sandwich immunoassay developed by Eli Lilly and Company (Indianapolis, IN). Briefly, a myostatin ELISA was performed using Mesoscale Discovery (MSD) plates with streptavidin-coated and pre-blocked wells that were incubated for 1-hour with biotinylated anti-myostatin capture antibody. Afterward, wells were aspirated and washed three times with TBST (Tris buffered saline containing 50 mmol/L Tris pH 7.40, 150 mmol/L NaCl, with 0.5 mL Tween 20/L). Next, 100 μL of recombinant myostatin standards (varying concentrations of myostatin protein in assay buffer consisting of 50 mmol/L HEPES, pH 7.40, 150 mmol/L NaCl, 10 mL/L Triton X-100, 5 mmol/L EDTA,5 mmol/L EGTA, and 0.1 mg/ml Heterophilic Blocking Reagent I (Scantibodies Laboratory Inc, Santee, CA)) were added to the wells to generate a standard calibration curve. Plasma samples were diluted in assay buffer and added to their respective wells, and plates were incubated for 1 hour at room temperature. Following aspiration, wells were washed 3 times with TBST, and 100 μL of conjugate antibody (ruthenium-labeled anti-myostatin detection antibody) were added to the wells for a 1-hour incubation at room temperature. Following aspiration, wells were washed 3 times with TBST, and the plate was developed using a MSD reader, which recorded ruthenium electrochemiluminescence. MesoScale Discovery (MSD) software and SigmaPlot version 8.0 were used for fitting the ELISA calibration curves as well as for final determination of serum levels.

### Transmission electron microscopy (TEM)

A small section (<5mg) of EDL was fixed with 10% glutaraldehyde and processed for transmission electron microscopy (TEM). TEM images were used to identify qualitative morphological differences in mitochondria.

### Immunohistochemistry

Immunostaining was performed on frozen cryostat sections of EDL. Hematoxylin and eosin (H & E) staining was performed as previously published [20]. Further, the sections were incubated for 30 minutes with succinate dehydrogenase (SDH, 0.4g sodium succinate, 0.04g NBT, 0.001mg phenazine methosulfate) in 0.1M Tris buffer at 37°C, then extracted with 30–90% acetone and rinsed with dH_2_O. Muscle fibrosis was also assessed in the frozen sections by Picrosirius red staining as previously described [18]. 10x images were taken using a Spot RT Color Camera System mounted on an inverted Nikon Diaphot 200 microscope (Nikon Instruments Inc, Melville, NY), 3 images per section, 3 sections per animals were analyzed. Thresholding was performed using Metamorph software (Molecular Devices, Sunnyvale, CA) and average intensity was reported.

### Statistics

Independent sample t-tests were used to compare means between CKD and NL rats. Pearson Product-Moment correlation was used to assess the relationship between myostatin to 8-OHdG data. Data are presented as mean ± SEM.

## Results

### The expression of myogenic and proteolytic factors are altered in CKD

**Fig 1:**
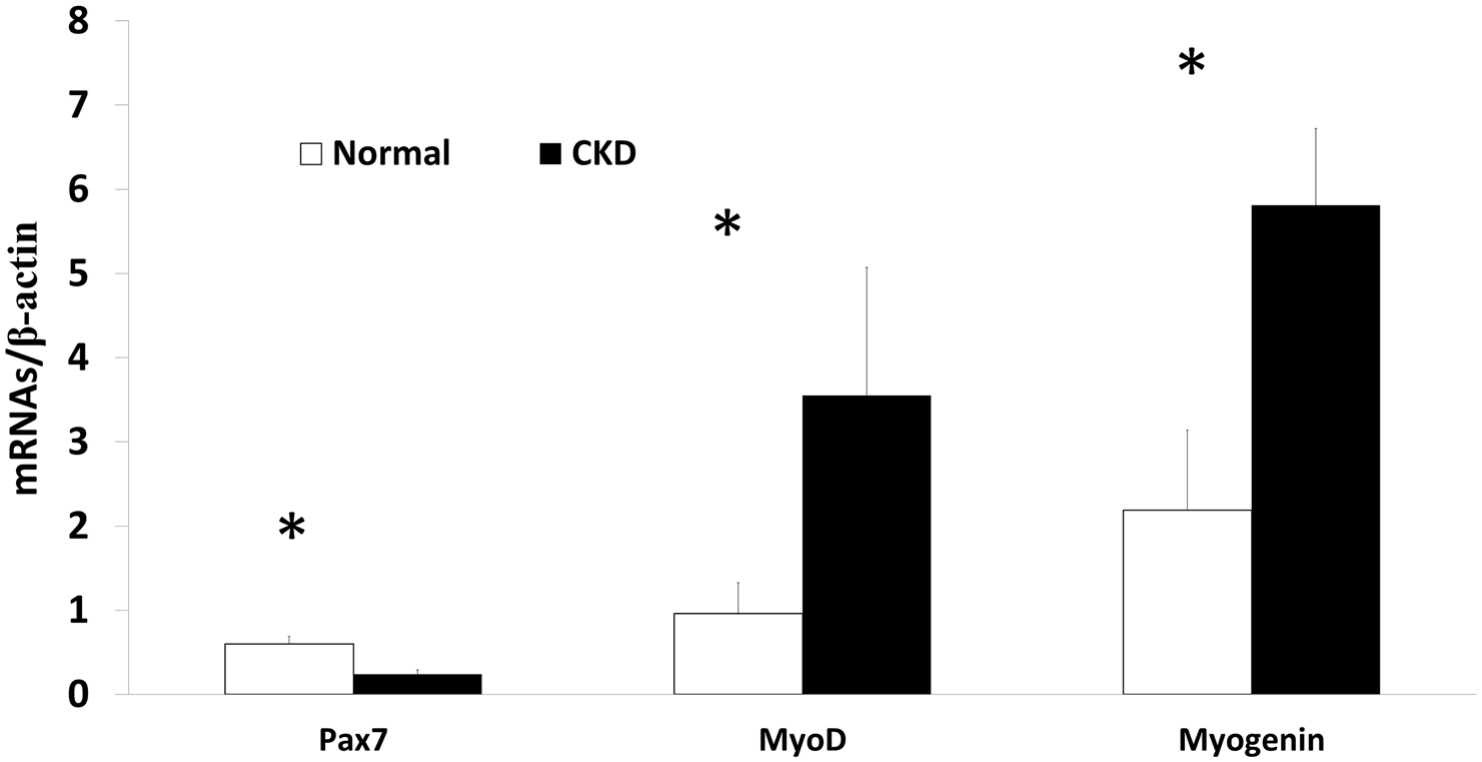
Myogenesis is altered in CKD. The expression of myogenic factors was determined by qRT-PCR in the EDL muscle from 35 weeks old normal littermates (NL) and chronic kidney disease (CKD) rats. In CKD there is increased activation and differentiation due to lower expression of Pax-7, and increased expression of MyoD and myogenin. Data are shown as mean ± SD (n = 6 rats each group). *P<0.05, NL vs. CKD.

**Fig 2:**
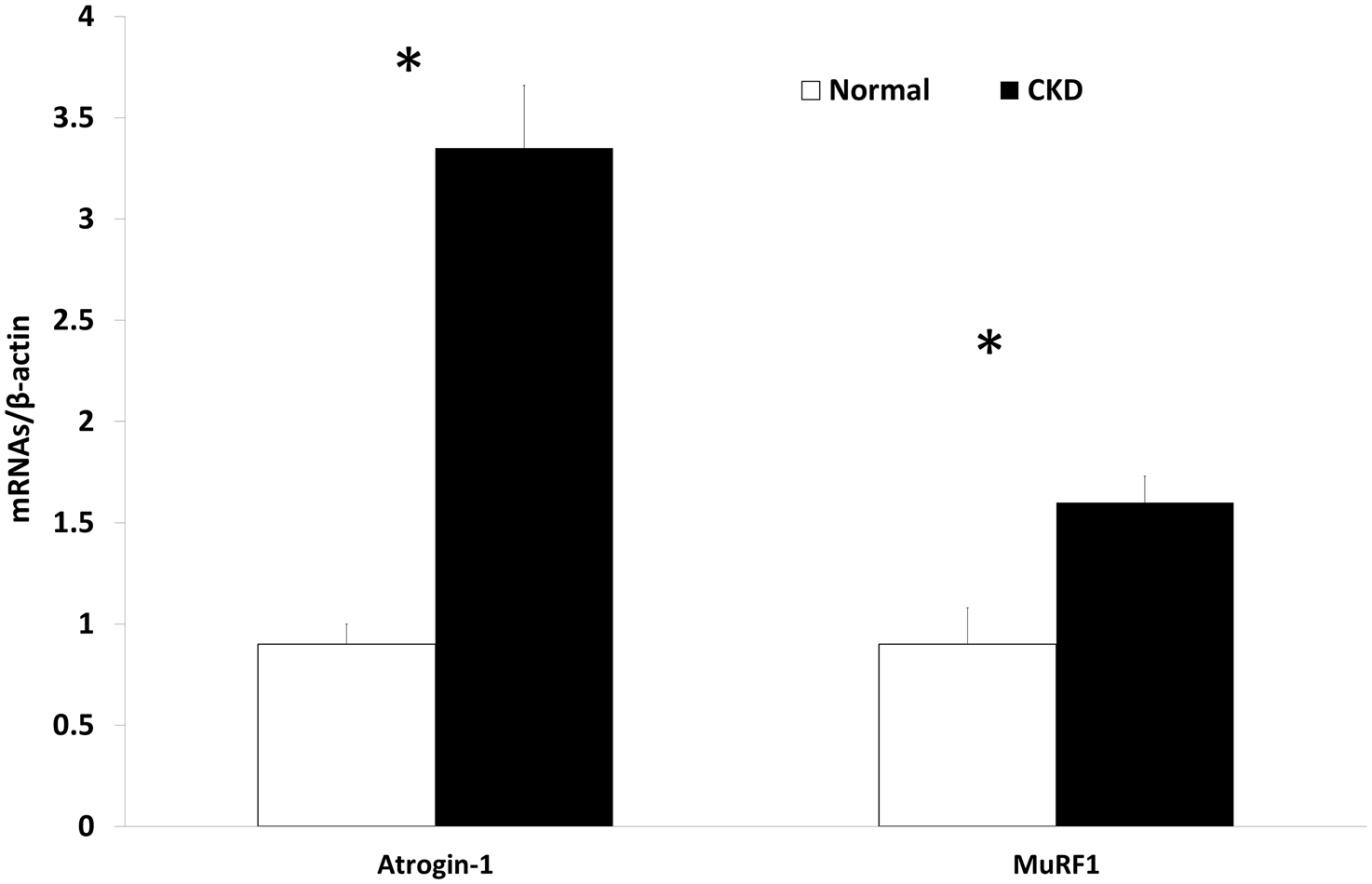
Proteolytic pathways in muscle are activated in CKD. Atrogin-1 and MuRF-1 expression was higher in the EDL of 35 week old chronic kidney disease (CKD) rats than normal littermates (NL), as determine by qRT-PCR. Data are shown as mean ± SD (n = 6 rats each group). *P<0.05, NL vs. CKD.

We assessed the gene expression of three myogenic regulatory factors (Pax-7, MyoD, and myogenin) from the EDL muscle isolated from 35 week old rats. The results demonstrated significantly lower expression of Pax-7 (reduced muscle stem cell quiescence) and higher expression of both MyoD (cell activation) and myogenin (cell differentiation) in CKD rats compared to that in NL rats (Fig 1). The expression of MiR-29b, a pro-myogenic and anti-fibrotic factor important during skeletal muscle cell differentiation [21], was significantly lower in CKD (0.96±0.11 vs. 0.60±0.31, p<0.02; NL vs. CKD, S1 Fig). However, there was no difference in the expression of the pro-fibrotic TGFβ between CKD and NL (0.75±0.2, 0.89±0.19, p = 0.26, respectively), nor was there a difference in the picrosirius stain for fibrosis (8.06±4.87 (CKD), 9.16±2.6 (NL), p = 0.51; S2 Fig). However, there was higher gene expression for muscle proteolytic markers Atrogin-1 and MuRF-1 in EDL from CKD compared to NL rats (Fig 2). H & E staining depicted sparse centralized nuclei per section in both NL and CKD rats (3.24± 1.3, 1.87±1.1, p = 0.09, respectively; S3 Fig). Taken together, these data suggest that CKD muscles are beginning the process of regeneration, with increased activation and differentiation, with concurrent increased proteolysis. These data also indicate that the previously observed fiber atrophy in this model is not due to fibrosis [16].

### CKD animals have increased myostatin and oxidative stress

**Fig 3:**
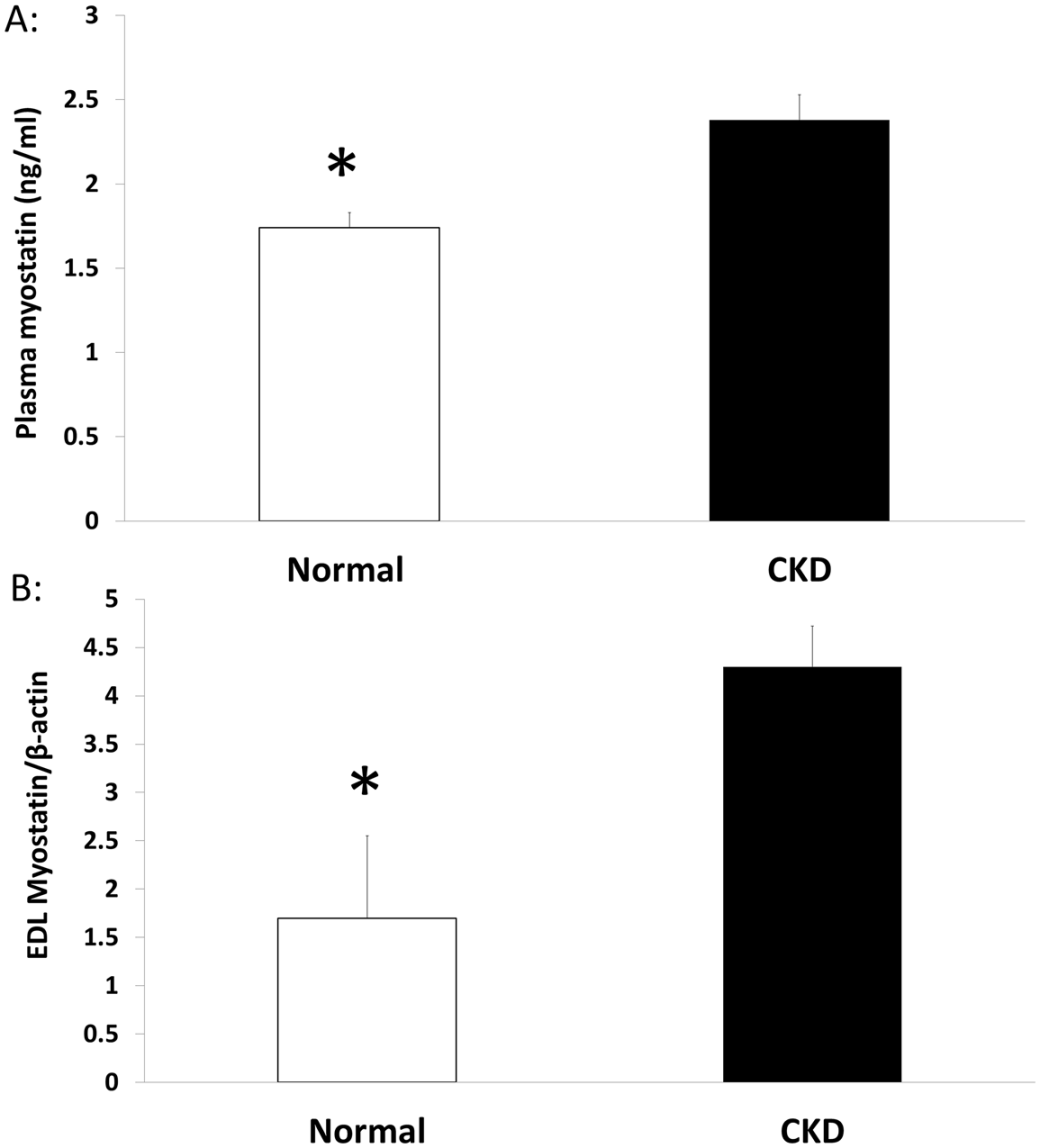
Myostatin is increased in plasma and muscle tissue in CKD. At 35 weeks, myostatin plasma levels were higher in chronic kidney disease (CKD) than normal littermates (NL) as determined by an ELISA (A). In EDL, via qRT-PCR analysis, myostatin expression was higher in CKD than NL at 35 weeks of age (B). Data are shown as mean ± SD (n = 6 rats each group). *P<0.05, NL vs. CKD.

**Fig 4:**
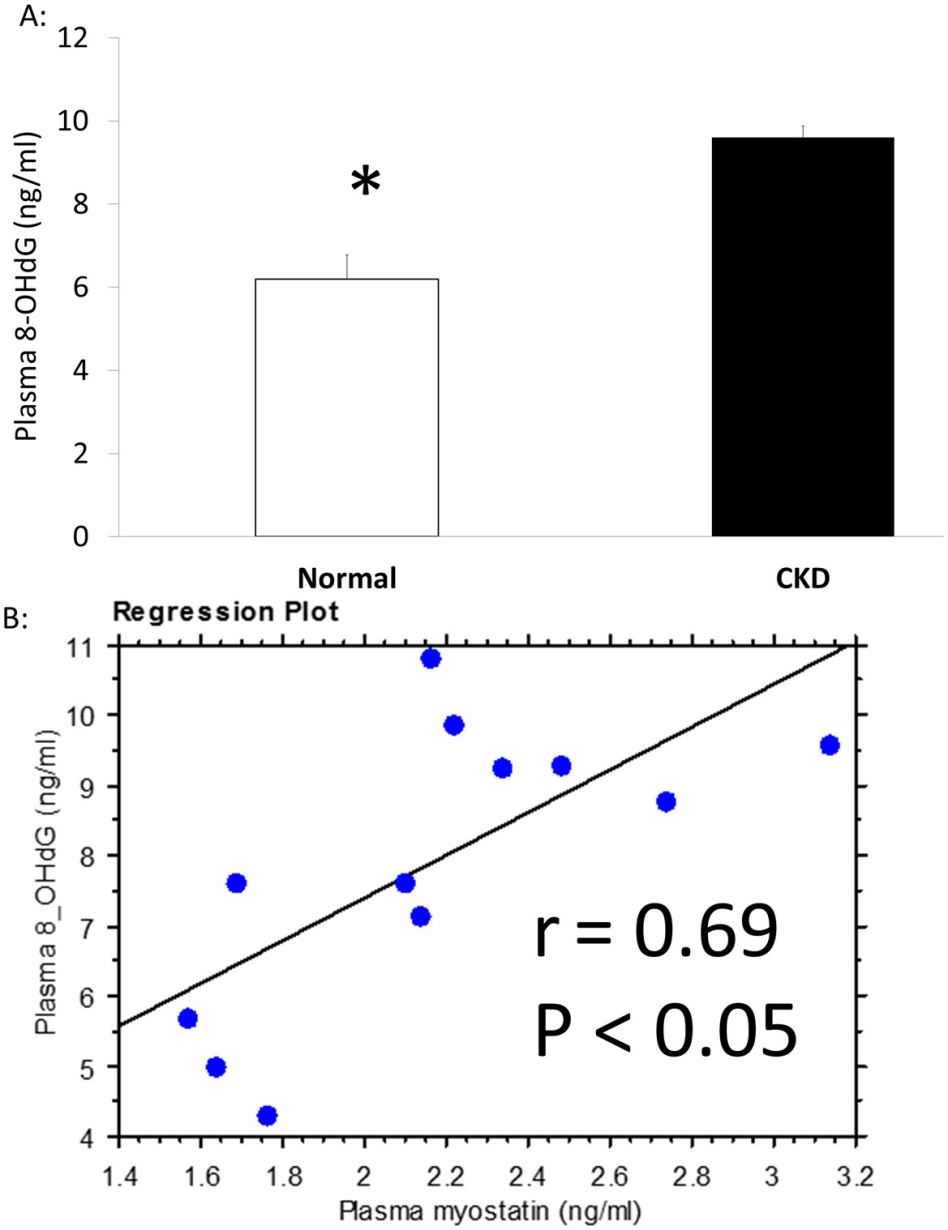
Oxidative stress is higher in CKD and is associated with plasma myostatin. 8-OHdG, an oxidative stress marker, was determined via an assay kit using blood collected from normal littermates (NL) and chronic kidney disease (CKD) rats at 35 weeks. Plasma levels of 8-OHdG were significantly higher in CKD (A) and correlated positively with plasma myostatin (B). Data are shown as mean ± SD (n = 8 rats each group). *P<0.05, NL vs. CKD.

The plasma levels of myostatin were significantly higher in CKD animals compared to NL animals (Fig 3A). This was corroborated by higher RNA expression of myostatin in EDL muscle tissue of CKD compared to NL (Fig 3B). In contrast, the myostatin receptor, activin 2b receptor was not different in CKD vs. NL animals (0.965 ±0.317, 0.815 ±0.137, p = 0.35; respectively; S4 Fig). Plasma 8-OHdG levels, a measure of DNA oxidative damage, were significantly higher in the CKD rats compared to normal rats (Fig 4A). The plasma levels of 8-OHdG and myostatin were positively correlated (r = 0.69, Fig 4B).

### Muscle from CKD animals show evidence of oxidative damage

**Fig 5:**
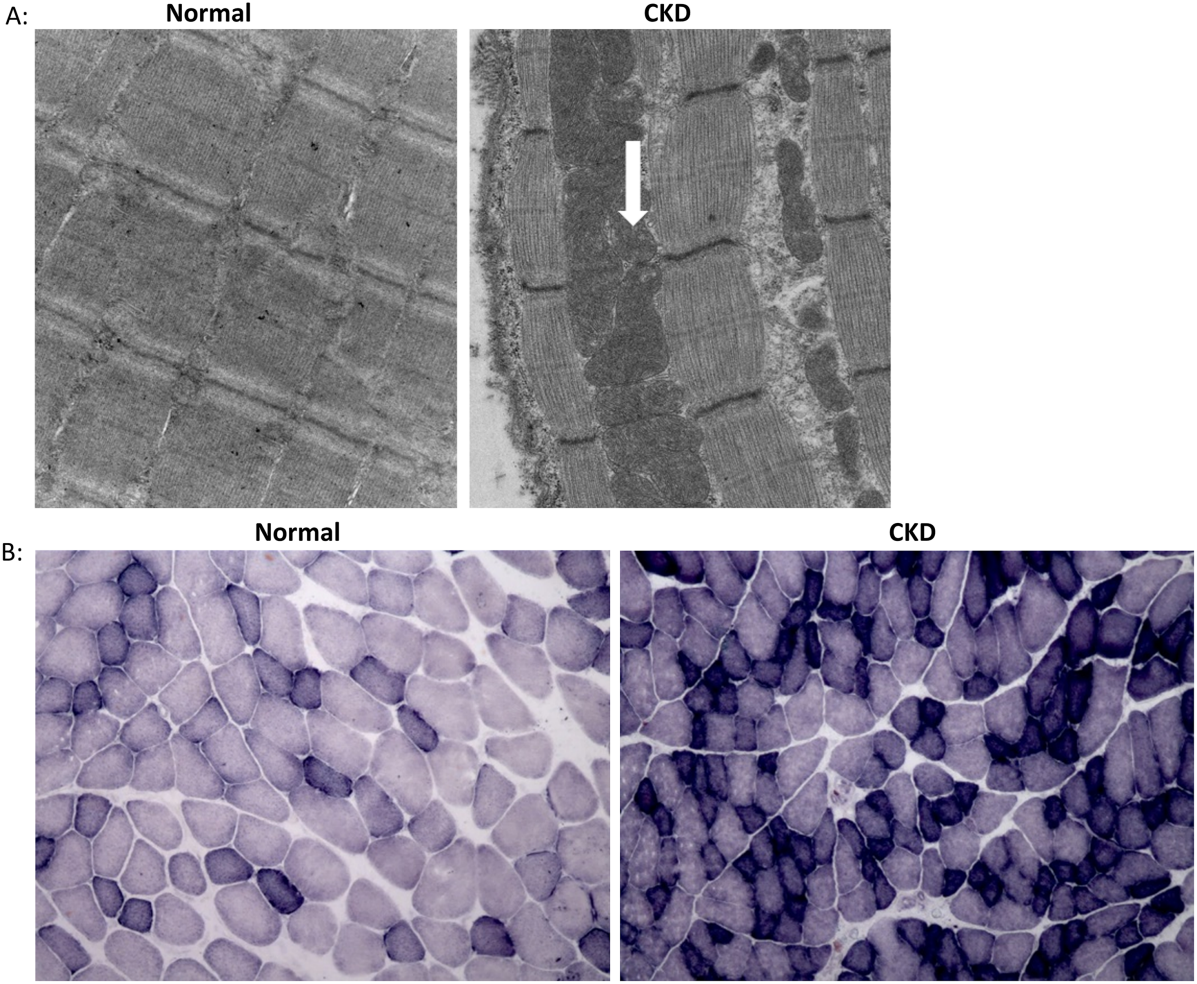
Mitochondria alterations in CKD. The EDL was collected from normal littermates (NL) and chronic kidney disease (CKD) rats at 35 weeks and processed for transmission electron microscope (EM) (40, 800 (print magnification, 23,000X direct magnification). The results demonstrated disrupted sarcomeres and engorged mitochondria (arrow) in CKD rats (upper right panel) compared to NL (upper left panel) (A). Qualitatively SDH immunostaining of the EDL was more prominent at 35 weeks of age in CKD (lower right panel) compared to that in NL (lower left panel) (B).

**Fig 6:**
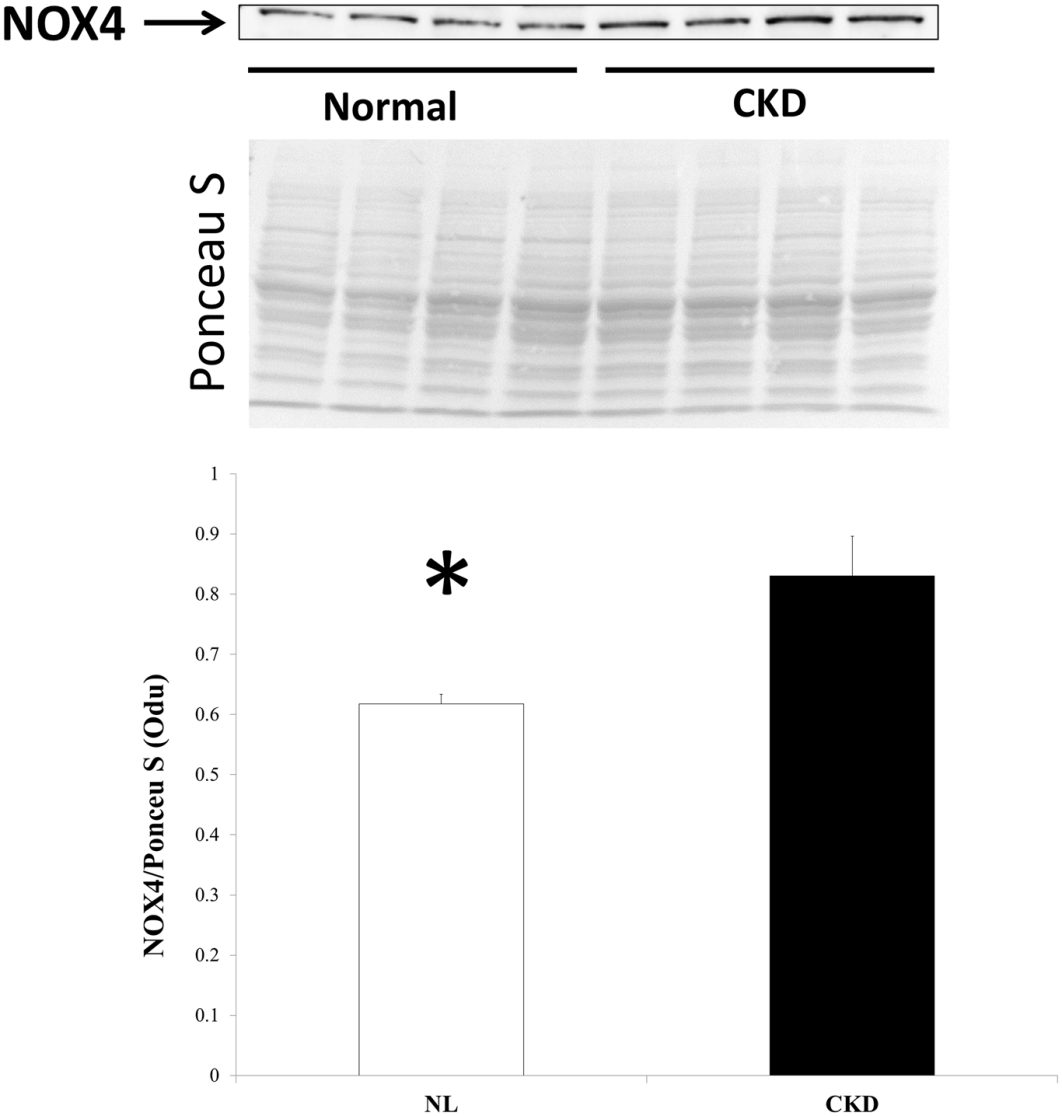
CKD skeletal muscle has higher protein expression of Nox4. Total protein was isolated from the EDL of 35 week old chronic kidney disease (CKD) and normal littermates (NL) rats and normalized to the total band of Ponceau S; Nox4 expression was significantly higher in CKD than NL. Data are shown as mean ± SD (n = 8 rats each group) with representative Ponceau S image used for normalization. *P<0.05, NL vs. CKD

**Fig 7:**
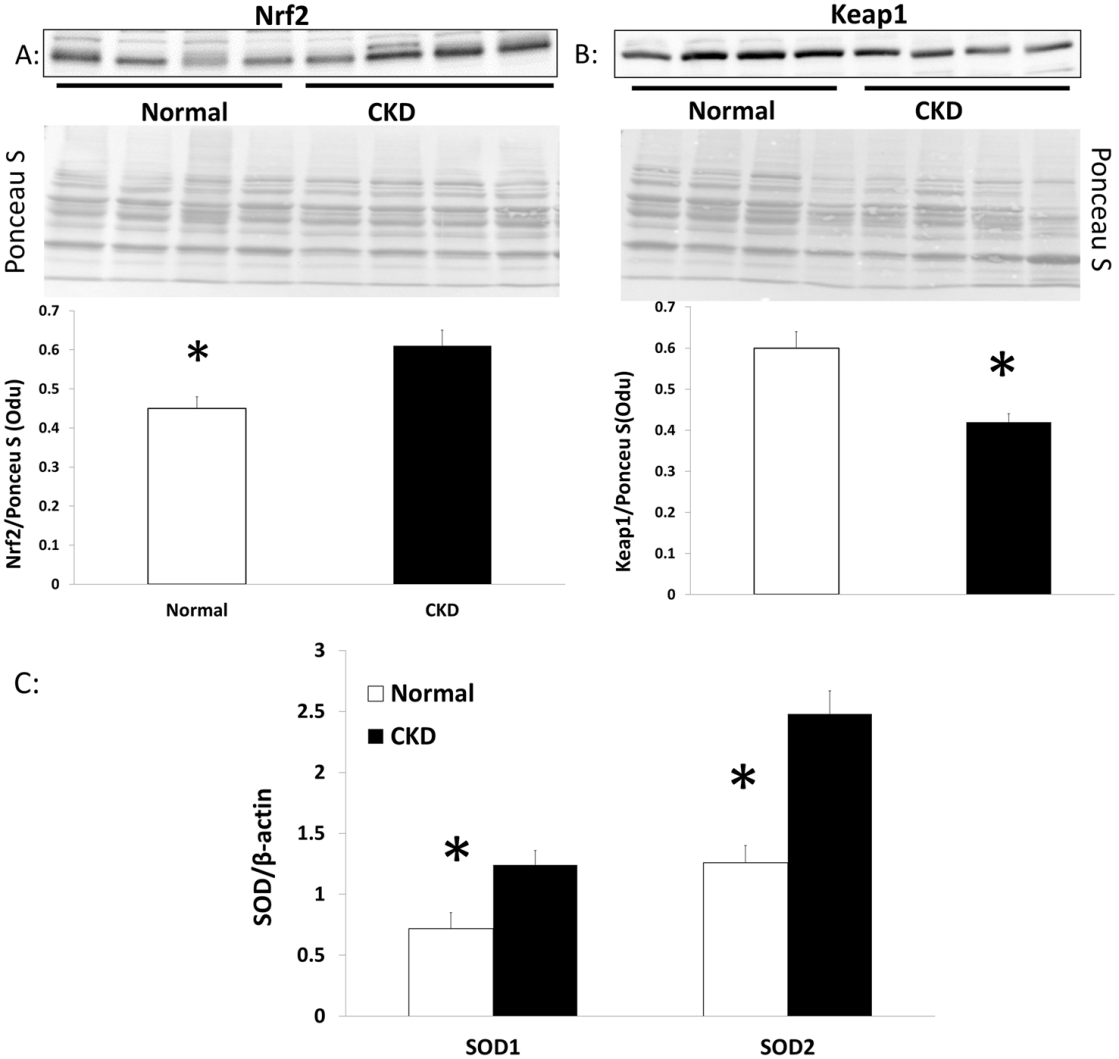
Antioxidant response in skeletal muscle is altered in CKD. Nuclear and cytosolic protein were isolated from the EDL of 35 week normal littermates (NL) and chronic kidney disease (CKD) rats and normalized to the total band of Ponceau S. In CKD, the expression of the antioxidant transcription factor, Nrf2 was significantly higher (Fig 7A), while the expression of the Nrf2 repressor, Keap1, was significantly lower (Fig 7B) as compared to NL. Data are shown as mean ± SD (n = 8 rats each group) with representative Ponceau S image used for normalization. The expression of SOD-1, 2 were determined by qRT-PCR in the EDL muscle from 35 weeks old NL and CKD rats. In CKD there is increased expression of SOD-1 and SOD-2 CKD (Fig 7C). Data are shown as mean ± SD (n = 6 rats each group). *P<0.05, NL vs. CKD, **P<0.001, NL vs. CKD.

Qualitative assessment by electron micrographs of one NL and one CKD animal demonstrated that muscle from CKD animals had disrupted sarcomeres, altered I-band structure (made of actin and titin), and engorgement of mitochondria (white arrow, right panel, Fig 5A). Histochemical staining of the EDL showed higher expression of succinate dehydrogenase (SDH) (Fig 5B) suggestive of oxidative stress within the CKD muscle. In support of these observations, western blotting demonstrated a significant, higher level of Nox4 expression in skeletal muscle from CKD compared to NL animals (Fig 6). A key transcription factor that upregulates multiple antioxidant pathways, Nrf2, was also significantly higher in skeletal muscle from CKD rats compared to that from NL rats (Fig 7A). In contrast, the expression of Keap1 (Nrf2 repressor) was significantly lower in CKD rats compared to NL (p<0.05, Fig 7B). Accordingly, the downstream target of Nrf2, the expression of superoxide dismutase (SOD) 1 and 2 was also increased in muscle from CKD animals compared to that from normal animals (SOD-1 p<0.05, SOD-2 p<0.001, Fig 7C).

## Discussion

Our results demonstrate that in this progressive CKD rat model, muscle atrophy occurs despite increased satellite cell activation and differentiation, which may be attributed to activation of proteolytic pathways. We demonstrated in CKD skeletal muscle tissue there is higher stem cell activation (decreased Pax-7, increase MyoD) and differentiation (myogenin) with the lower expression of a pro-myogenic factor, miR-29. Despite the increased activation, there is a disconnect with virtually no centralized nuclei to demonstrate that regeneration is taking place. Similar patterns of increased activation and differentiation have been found in rodent models of aging; this pattern was suggested to reflect a regenerative drive from disrupted tissue integrity related to sarcopenia [22]. However, in a mouse models of surgically-induced CKD MyoD, myogenin and Pax-7 were all significantly reduced ranging from 20–80% less expression [23]. There are clear differences in transcription factor expression between the current model and the surgically-induced models, but it is not clear what the underlying differences are or how the differences impact skeletal muscle. Models comparisons could be explored in the future to determine factors associated with these differences. Further, there was higher proteolytic activity demonstrated by the expression of Atrogin-1 and MuRF-1. Given we have previously shown reduced skeletal muscle fiber cross-sectional area and strength in this same animal model (16), it appears that the increased myogenic regulatory factor expression is insufficient to overcome catabolic processes. Thus, in CKD, muscle loss may be accelerated due to an imbalance between regenerative and proteolytic processes. Skeletal muscle loss associated with CKD manifestations is a complex problem that has clinical implications related to quality of life, morbidity and mortality.

The increased muscle degradation that contributes to skeletal muscle atrophy in CKD could be the result of increased oxidative stress. Oxidative stress is the net balance of oxidant production to antioxidant defense, and is central to the pathogenesis and progression of CKD [24, 25]. Oxidative stress during aging is considered to be a major contributor of muscle atrophy [7, 8], with mitochondria potentially being the primary producer of oxidants [26]. Our results indicate higher oxidative stress both systemically and in muscle of CKD rats evidenced by elevated plasma levels of DNA oxidative stress marker 8-OHdG, qualitative presence mitochondrial derangement, increased mitochondrial activity (complex II via SDH stain), and increased Nox4 protein expression in skeletal muscle. Our mitochondrial activity via SDH is in opposition to downregulation of citrate synthase activity and mitochondrial biogenesis gene expression [27]. This discrepancy may be the result of utilized animal models, stage of kidney disease, and/or rat versus mouse and requires further exploration. The isoform Nox4 is an O_2_ sensor in skeletal muscle that regulates oxidant production and ensuing oxidative stress [28]. Increased Nox4 is thought to increase sarcoplasmic reticulum (SR) Ca^2+^ leak [29] by oxidizing the ryanodine receptor and lowering intracellular calcium, which has been shown to cause muscle weakness in aging and cancer cachexia [30, 31]. Further, upregulated Nox4 causes sarco/endoplasmic reticulum Ca^2+^-ATPase (SERCA) oxidation [32], which decreases calcium uptake the SR and reduces the efficiency of skeletal muscle relaxation [33]. Such inability to buffer intracellular calcium may alter skeletal muscle contraction, and may explain our increase in half-relaxation time in these animals as previously reported [16]. We found in CKD there is higher Nox4 protein expression despite downregulated Nox4 RNA expression. This paradoxical relationship between Nox4 RNA and protein has been found in endothelial cells and vascular smooth muscle cells [34, 35]. Although the underlying mechanism is not clear, it has been suggested that alterations of transcription, translation and the stability of mRNA or protein may be involved. Nox4-generated ROS has also been found to increase myoblast proliferation and differentiation [36]. However, this was in a C_2_C_12_ cell culture model with short-term exposure. It is not clear how Nox4 expression mediates cell proliferation or differentiation in a CKD model. Nox4-generated ROS may be similar to that of exercise-induced H_2_O_2_; where periodic increases are beneficial while chronic exposure is detrimental [37]. While Nox4 is increased, further investigation is needed to determine what its role underlying skeletal muscle dysfunction is in CKD.

Increased oxidant production can be offset by simultaneous up-regulation of the antioxidant system. Under homeostatic conditions, the master antioxidant transcriptional factor, Nrf2 is maintained in the cytoplasm by Keap1. Upon production of reactive oxygen species, Keap1 is degraded and allows Nrf2 to translocate to the nucleus to activate downstream antioxidant targets. In CKD, Nrf2 is deficient in the kidney and this has been shown to lead to increased oxidative stress [38]. We therefore anticipated a similar response in the skeletal muscle of Nrf2 being down-regulated with an up-regulation of Keap1. To the contrary, we found increased expression of antioxidant transcription factor as demonstrated by an increase in Nrf2 and a decrease in Keap1. Furthermore, the expression of SOD1 and 2, the downstream target gene of Nrf2, are increased in muscle from CKD rats compared to that in normal rats. The increased antioxidant production, however, was insufficient to overcome oxidant production, demonstrated by increased plasma 8-OHdG and apparent mitochondrial damage on TEM imaging. It is not clear how tissue-specific antioxidant responses change with disease progression/severity or how they are influence by therapeutic interventions.

Oxidative stress may also impair skeletal muscle by activating myostatin expression and inducing skeletal muscle atrophy by activating autophagy pathways [9], and increased FoxO-mediated transcription of atrogenes, Atrogin-1 and MuRF-1 [10]. In our CKD model, we found higher tissue myostatin expression and plasma myostatin levels; plasma myostatin levels were also significantly correlated with a marker for oxidative stress, 8-OHdG (r = 0.69). Myostatin, is a negative regulator of muscle mass that has been shown to increase in a number of pathologies, including, cancer cachexia [39], heart failure [40] and chronic obstructive pulmonary disease [41]. Myostatin represents one pathway that regulates the balance between catabolic and anabolic processes. Clinically, myostatin is significantly upregulated in patients with CKD [42]; CKD patients with high myostatin levels are seven times more likely to have lower grip strength [43]. In a pre-clinical model of kidney disease (5/6 nephrectomy), there was increased myostatin expression and muscle atrophy which was improved with an anti-myostatin peptide [14, 44].

Myostatin and oxidative stress have both been found to influence muscle atrophy by altering antioxidant response and increasing proteolytic activity [12, 45]. Increased myostatin levels have been shown to induce oxidative stress and increase SOD in skeletal muscle cells [11]. In our CKD model which demonstrated muscle atrophy [16], there was higher expression of SOD-1, SOD-2, Atrogin-1 and MuRF-1. We found similarities with both increased myostatin and SOD mRNA expression, but future studies will be needed to confirm oxidative stress caused atrophy. Atrogin-1/MuRF-1 translational activity was increased 20-fold in diabetic rats via suppression of phosphatidylinositol 3 kinase activity (PI3K), which caused significant muscle atrophy [46]. Furthermore, Atrogin-1/MuRF-1 can be regulated by FoxO transcription factors; in a FoxO1 knockout mouse that underwent a 2-stage nephrectomy demonstrated 70% suppression of Atrogin-1/MuRF-1, suppressed proteolysis and no muscle atrophy [47]. The increase in proteolysis is in line with the dysfunctional skeletal muscle in our CKD model. It is not clear how myostatin or proteolysis differ among different kidney injury model, but should be further explored in a progressive CKD model. Thus, inhibition of myostatin may have a positive impact on muscle size, but given the complexity of pathologic changes observed, including oxidative stress, this may not translate to improved muscle function.

In summary, we demonstrated in a slow progressing, naturally occurring model of CKD in animals with severe kidney disease there is enhanced activation of skeletal muscle stem cells, increased myostatin expression and downstream activation of the atrogenes, Atrogin-1 and MuRF1. Similarly, the presence of mitochondrial damage, increased Nox4 and DNA oxidative damage marker (8-OHdG) demonstrates an inadequate increase of antioxidant activation to overcome oxidant production. Augmentation of the myogenic and antioxidant responses through treatments such as anti-myostatin therapies and/or anti-oxidants may prove effective to prevent/slow progressive sarcopenia in CKD. The clinical importance of maintaining skeletal muscle mass is evident both in response to disease resistance as skeletal muscle size, quality and/or performance are involved in maintaining and/or improving physical function and quality of life.

## Supporting Information

S1 Fig MiR-29 pro-myogenic factor is lowered in CKD. The expression of MiR-29 was determined by qRT-PCR in the EDL muscle from 35 weeks old normal littermates (NL) and chronic kidney disease (CKD) rats. In CKD there is lower expression of miR-29b compared to normal. Data are shown as mean ± SD (n = 6 rats each group). *P<0.05, NL vs. CKD.
(TIF)

S2 Fig Representative Picrosirius Stain Image. There was no there a difference picrosirius stain for fibrosis (8.06±4.87 (chronic kidney disease (CKD)), 9.16±2.6 (normal littermates (NL)), p = 0.51). Representative photos are provided for CKD (left) and NL (right).
(TIF)

S3 Fig Representative H & E Image. There was no there a difference in centrally-located nuclei between normal littermates (NL) and chronic kidney disease (CKD) rats (3.24± 1.3, 1.87±1.1, p = 0.09; respectively). Representative photos are provided for CKD (left) and NL (right).
(TIF)

S4 Fig Activin 2b receptor expression is not different between CKD and normal animals. The expression of activin 2b was determined by qRT-PCR in the EDL muscle from 35 weeks old normal littermates (NL) and chronic kidney disease (CKD) rats. There is no difference in expression between CKD and NL (0.965 ±0.317, 0.815 ±0.137, p = 0.35; respectively).
(TIF)
